# Supplementary material for: Barriers and Facilitators to Smart Healthcare Adoption Among Chinese Patients with Cardiovascular Disease and Their Caregivers: A Qualitative Study
Source: Healthcare (Basel). 2025 Nov 12;13(22):2881. doi: 10.3390/healthcare13222881 (PMC12652973; doi:10.3390/healthcare13222881)
Supplement: Supplementary file 1 [file healthcare-13-02881-s001.zip › healthcare-3860647-supplementary.pdf]

Supplementary Table S1. Interview guide.

Topical Area	Interview Question	Purpose of the Question
Exploration of health information behaviors	(1) What are the main channels through which you obtain health knowledge? Why do you prefer these channels?<br>(2) When was the last time you actively inquired about health knowledge? What prompted you to do so?	To understand participants' existing health information literacy and seeking habits, establishing a baseline context for their adoption of new tools.
Experiences with smart healthcare use	(3) Could you describe any smart devices or mobile applications you have used in managing your own (or your family member's) cardiovascular health?<br>If yes: Please describe in detail the one or two tools you use most frequently.<br>If no: What are the reasons that have prevented you from trying such tools?	To investigate the specifics of participants' exposure to and use of smart healthcare tools, distinguishing between users and non-users to objectively understand their experiences or barriers.
Perceived benefits and empowerment	(4) When using these tools, what aspects do you find most helpful for daily health management (or for managing the health of your family)?<br>(5) Has this process impacted your confidence in health management? In what ways?	To delve into the positive impacts and value derived from smart healthcare, exploring how these tools promote self-efficacy and changes in self-management behaviors.
Challenges and coping strategies	(6) What challenges or issues have you encountered while using these programs?<br>(7) How did you resolve these issues?	To identify obstacles encountered during use and understand participants' spontaneous coping strategies and unmet needs.
Willingness for sustained use and conclusion	(8) What factors do you think would motivate you to persist in using a health management tool?<br>(9) Are there any other experiences or feelings related to health management that you would like to share?	To explore factors motivating long-term use and to capture any unpredicted key information with a concluding open-ended question.

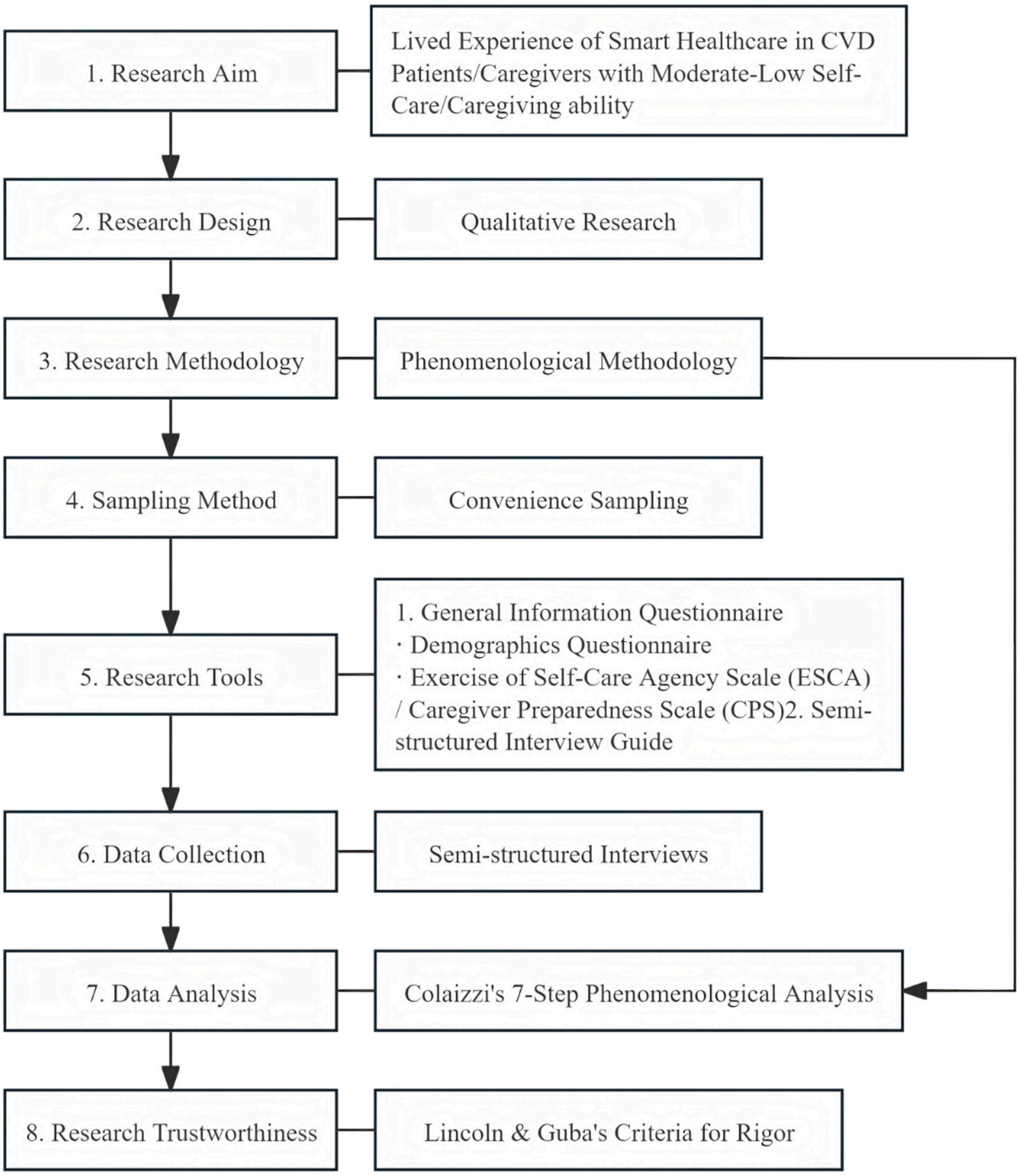

Supplementary Figure S1. Study Design Diagram.
